# Dynamics of a Cytokine Storm

**DOI:** 10.1371/journal.pone.0045027

**Published:** 2012-10-01

**Authors:** Hao Hong Yiu, Andrea L. Graham, Robert F. Stengel

**Affiliations:** 1 Department of Chemical and Biological Engineering, Princeton University, Princeton, New Jersey, United States of America; 2 Department of Ecology and Evolutionary Biology, Princeton University, Princeton, New Jersey, United States of America; 3 Department of Mechanical and Aerospace Engineering, School of Engineering and Applied Science, Princeton University, New Jersey, United States of America; UMIT, Austria

## Abstract

Six volunteers experienced severe inflammatory response during the Phase I clinical trial of a monoclonal antibody that was designed to stimulate a regulatory T cell response. Soon after the trial began, each volunteer experienced a “cytokine storm”, a dramatic increase in cytokine concentrations. The monoclonal antibody, TGN1412, raised serum concentrations of both pro- and anti-inflammatory cytokines το very hiγh values during the first day, while lymphocyte and monocyte concentrations plummeted. Because the subjects were healthy and had no prior indications of immune deficiency, this event provided an unusual opportunity to study the dynamic interactions of cytokines and other measured parameters. Here, the response histories of nine cytokines have been modeled by a set of linear ordinary differential equations. A general search procedure identifies parameters of the model, whose response fits the data well during the five-day measurement period. The eighteenth-order model reveals plausible cause-and-effect relationships among the cytokines, showing how each cytokine induces or inhibits other cytokines. It suggests that perturbations in IL2, IL8, and IL10 have the most significant inductive effect, while IFN-*γ* and IL12 have the greatest inhibiting effect on other cytokine concentrations. Although TNF-*α* is a major pro-inflammatory factor, IFN-γ and three other cytokines have faster initial and median response to TGN1412 infusion. Principal-component analysis of the data reveals three clusters of similar cytokine responses: [TNF-*α*, IL1, IL10], [IFN-*γ*, IL2, IL4, IL8, and IL12], and [IL6]. IL1, IL6, IL10, and TNF-*α* have the highest degree of variability in response to uncertain initial conditions, exogenous effects, and parameter estimates. This study illuminates details of a cytokine storm event, and it demonstrates the value of linear modeling for interpreting complex, coupled biological system dynamics from empirical data.

## Introduction

Cytokines are signaling peptides, proteins, or glycoproteins that are secreted by many cell types, including immune, epithelial, endothelial, and smooth muscle cells. They either enhance or inhibit inflammation in response to pathogens, “non-self” molecules, and toxins. Cytokines allow context-dependent communication within the body [1], [2], [3], [4], [5]. If the interactions that lead to cytokine production are destabilized, a "cytokine storm" (or hypercytokinemia) can result, producing unbridled inflammation within tissues and key organs. Cytokine storms are associated with sepsis and septic shock [6], influenza, acute respiratory distress [7], host response to blood transfusion or bone marrow transplantation, and toxic response to medication. They have been implicated in the 1918 Spanish flu pandemic, the 2003 severe acute respiratory syndrome (SARS) outbreak, and the H5N1 avian influenza infections first recognized in 1987 [8], [9].

Activation of CD4+ (helper and regulatory) T cells normally requires two signals, one from an antigen-MHC complex to the T-cell receptor, and a concurrent co-stimulatory signal to a cell surface receptor, CD28, that is provided by antigen-presenting cells [10], [8], [11]. TGN1412 is a genetically engineered CD28 antibody agonist that can activate T cells without a co-stimulatory antigen signal [12], [11]. The drug’s manufacturer saw a potential application in patients with chronic lymphocytic leukemia, whose T-cell population had been destroyed by chemotherapy along with the cancerous B cells. They also saw potential applications in boosting regulatory T cells to treat autoimmune and inflammatory diseases such as rheumatoid arthritis, where effector T cells become overactive and pathogenic [12].

With efficacy demonstrated in animal models, Phase I clinical trials were scheduled for testing in humans. In March 2006, six healthy male volunteers received TGN1412, and two volunteers received a placebo. Great care was taken in establishing a dosage that was deemed to be safe in humans [12]. Within an hour of infusion, the six who received the drug experienced adverse effects while those who received placebo did not. The drug’s recipients had headaches, muscle pain, nausea, diarrhea, decreased blood pressure, and increased heart rate, all indications of systemic inflammatory response syndrome (SIRS) [13], [11]. Severe depletion of lymphocytes and monocytes occurred four hours after drug infusion and continued until the fourth day. C-reactive protein, serum creatinine, and neutrophil concentrations increased well above normal levels during the same time period. Eventually, all six patients experienced multi-organ failure, with infiltrates in the lung, intravascular coagulation, renal failure, and lung injury [11]. Critical care and subsequent treatment included dialysis, mechanical ventilation, and, in one case, surgery to counter peripheral ischemia [11], [14]. The periods of illness extended beyond one month and may have induced permanent damage for all patients. Biological explanations of the event are offered in [15], [16], [17], [18], [19], [20]. Infection, underlying disease, or endotoxins did not cause this unique event; hence, it allowed unusual insight into the course and impact of immune-mediated cytokine storms. The analysis presented here particularly focused on time-dependent coupled interactions among the nine cytokines measured. The ability of the model to capture dynamics suggests that the nonlinearities and complexities of cytokine storms may not prove an insurmountable problem for mathematical biologists.

## Data and Methods

We have analyzed cytokine levels that were collected during the first five days of the 2006 TGN1412 clinical trial. TGN1412 dosage was 0.1 mg per kg of body mass, which averaged 79.7 kg for the six patients. The drug was infused at a rate of 2 mg/min over an average time of 3.97 min. The infusion time was short compared to measurement intervals and the time scale of response. Effects of different infusion durations are estimated from the derived dynamic model in the sequel.

When the patients began to experience adverse side effects, clinicians treated the subjects with corticosteroids, chlorpheniramine, acetaminophen, ondansetron, metaraminol, methylprednisolene, and an anti-IL2 receptor antagonist antibody. All subjects received aggressive individualized treatment while in intensive care. Consequently, the recorded cytokine histories reflect not only the natural reactions of the subjects but their response to therapy.

Concentrations of Tumor Necrosis Factor (TNF)-*α*, Interferon (IFN)-*γ*, Interleukin (IL) 10, IL8, IL6, IL4, IL2, IL1, and IL12 were measured eight hours before drug infusion, and 1, 4, 26, and 40 hours post-infusion. Clinicians then took measurements every six hours through day 4 and daily until day 10. These values were linearly interpolated to points tabulated at 6-hr intervals through day 5 for our analysis. TNF-*α*, IFN-*γ*, IL1, IL2, and IL8 are generally characterized as pro-inflammatory cytokines, IL4 and IL10 as anti-inflammatory cytokines, and IL6 and IL12 as either, depending on the signaling pathway [21].

Ordinary differential equations are often used to describe population dynamics of immune cells, pathogens, and signaling proteins [9], [22], [23], [24]. Here, we present linear, time-invariant models whose parameters are estimated from the median time series data for the six TGN1412 clinical subjects [11]. The models describe the evolution of nine cytokine concentrations without regard to the cells that secrete or are affected by them; cellular sources and sinks reported in the literature are discussed in a later section. Separate second-order equations for the concentrations and rates-of-change of each cytokine are constructed, and best-fit time constants are found by numerical search. All nine cytokines are then analyzed concurrently in an eighteenth-order system. A unified search over all of the higher-order system’s parameters improves the fit to the measured variables and provides an integrated model of the cytokine storm event. The coupled model illustrates multi-class interactions among the cytokines, identifies response modes and mode shapes (i.e., eigenvalues and eigenvectors), and reveals similarities in principal components. The data set, differential- and difference-equation models, and numerical search algorithm are described. The effects of uncertainty in initial conditions, exogenous effects, and parameter estimates are evaluated using a stochastic extension of the linear model.

**Figure 1:**
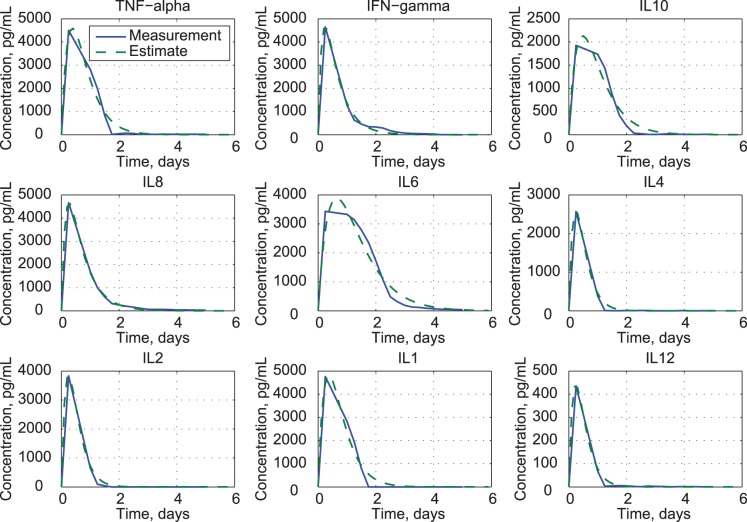
Comparison of clinical trial data [11] and estimates from uncoupled second-order models of cytokine response.

Normal cytokine concentrations range from 3.7 pg/mL (IFN-*γ* and IL10) to 48 pg/mL (IL1) or less, several orders of magnitude below the maximums shown in Fig. 1. The points identified as “Measurement” in Fig. 1 are the median values for all six patients referenced to the baseline at infusion time, as presented in Fig. 3 of [11]); inter-quartile error bars, which often span the measurement range, also are shown in this figure. Supplementary material shows considerable patient-to-patient variation in cytokine profiles. Signal saturation limits for the cytometric bead array immunoassays were 5,000 pg/mL; thus, higher cytokine values could not be detected. Because individual signals frequently reached saturation limits during the first few days of the event, estimates of several peak cytokine levels (particularly IL6, IL10, TNF-*α*, and IFN-*γ*) may be conservative. The median rather than the mean (or average) for the six individuals is presented because saturation would bias the mean computation for the original data.

## Results

### Modeling the Response of Individual Cytokines

The growth and decay of an individual cytokine’s response to its initial state is first represented by a second-order, linear, time-invariant ordinary differential equation. Denoting the serum concentration by *x*_1_(*t*) and its rate of change by *x*_2_(*t*),
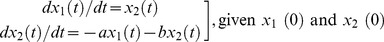
(1,2)


Acceleration of the cytokine concentration is represented by 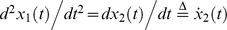. In vector-matrix form, the differential equation is expressed as.
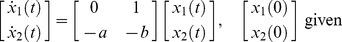
(3a)or, with the (2×1) state vector, **x**(*t*), and (2×2) stability matrix, **A**, in the general form,
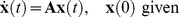
(3b)


The initial concentration, *x*_1_(0) = 0, is referenced to the cytokine’s basal level, and the initial rate of change, *x*_2_(0), is stimulated by the TGN1412 infusion. *a* and *b* are positive constants that express the sensitivity of the cytokine’s acceleration,*x*_2_(*t*), to concentration and rate of change. For this analysis, *a*, *b*, and *x*_2_(0) are determined by least-squares fit to the trial’s median response. The effects of clinical treatment are subsumed in the values of *a* and *b*.

The cytokine’s response modes are characterized by the eigenvalues, *λ*_1_ and *λ*_2_ (rad/day), of **A**, which are the roots of the characteristic equation,
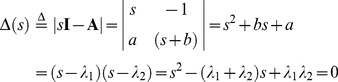
(4)


Consequently, eq. 3 can be expressed as.
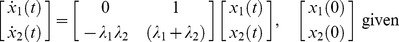
(5)


With real-valued eigenvalues, the response time constants, *τ*_1_ and *τ*_2_ (days), are the negative inverses of *λ*_1_ and *λ*_2_, and the two response modes reflect exponential growth or decay. If the eigenvalues are complex, the roots are complex conjugates (*σ* ± *jω*), and they define a single mode of response with natural frequency, *ω_n_*, damping ratio, *ζ*, and period, *P*. These are, respectively,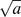, 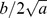, and 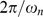. For *ζ* <1, the mode is underdamped and oscillatory.

The (2×1) eigenvectors, **e** *_i_*, of **A** describe the relative response of each state element in the mode defined by *λ_ i_*. They are solutions to the *n^th^*-order equation,
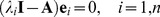
(6)where *n* = 2 for the second-order model. The equation can be multiplied by an arbitrary constant, *α*, without changing the equality, and so the absolute magnitude and phase of **e** *_i_* have no significance. For the second-order system (eq. 5), the eigenvectors can be expressed as
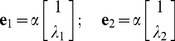
(7,8)


The system’s differential equation is converted to a difference equation to facilitate parameter identification. The state can be propagated from one sampling instant, *t_k_*, to the next, *t_k_*_+1_, using a (2×2) state-transition matrix, 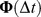, where 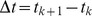, and 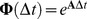. Hence, equation 3b is equivalent to.
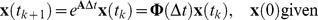
(9)


Because 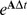 is the inverse Laplace transform of 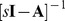, the second-order difference equation can be expressed as,
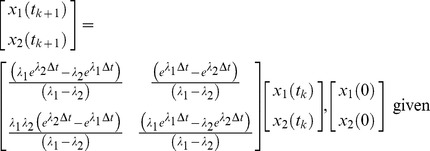
(10)


The extrapolations of eq. 9 and 10 are exact, and the identity of the continuous-time model is not lost. Therefore, the discrete-time model is employed in the numerical search for *a*, *b*, and *x*_2_(0), or, equivalently, *λ*_1_, *λ*_2_, and *x*_2_(0).

Model parameters are chosen to minimize the error between the cytokine concentration propagated by eq. 10, *x*_1_(*t_k_*), and the clinical trial measurements, *z*(*t_k_*). Positive and negative errors are equally unacceptable; hence a positive-definite quadratic cost, *J*, that sums the squares of the errors, *ε*(*t_k_*), at 6-hr intervals is an appropriate metric:
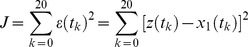
(11)


*J* is minimized using the downhill simplex (or Nelder-Mead) algorithm, as implemented in MATLAB’s *fminsearch* [25]. The iteration for each cytokine model is terminated when its error cost falls below an acceptable tolerance, as defined by the default settings for the MATLAB algorithm.

Functional minimization of eq. 11 for each cytokine generates nine sets of *λ*_1_, *λ*_2_, and *x*_2_(0) (Table 1). The initial rate of change in concentration represents each cytokine’s response to drug input. The eigenvalues are negative; hence, all modes are stable. The five pro-inflammatory cytokines are the fastest to respond to TGN1412 infusion. Time constants of response range from 0.14 to 0.65 days. The responses also can be modeled as critically and overdamped oscillations (*ζ* ≥1), with periods, *P*, from 1.5 to 4.1 days. The eigenvectors for each mode are determined by inspection from Table 1 and eq. 7 and 8.

**Table 1:** Eigenvalues, Time Constants, Periods, Damping Ratios, and Initial Rates of Change for Uncoupled, Second-Order Cytokine Models.

Component	*λ*_1_, d^−1^	*λ*_2_, d^−1^	*τ*_1_, d	*τ*_2_, d	*P*, d	*ζ*, -	*x*_2_(0), pg/mL-d
**TNF-** ***α***	−2.63	−2.63	0.38	0.38	2.39	1	32821
**IFN-** ***γ***	−7.21	−2.05	0.14	0.49	1.63	1.2	55328
**IL10**	−2.08	−2.08	0.48	0.48	3.02	1	12047
**IL8**	−6.71	−1.84	0.15	0.54	1.79	1.22	50804
**IL6**	−1.55	−1.55	0.65	0.65	4.05	1	16437
**IL4**	−4.17	−4.17	0.24	0.24	1.51	1	29489
**IL2**	−4.08	−4.08	0.25	0.25	1.54	1	42780
**IL1**	−2.71	−2.71	0.37	0.37	2.32	1	35535
**IL12**	−4.13	−4.13	0.24	0.24	1.52	1	4947

Uncoupled cytokine models fit the measured cytokine responses reasonably well (Fig. 1), but they reveal nothing about cytokine interactions. Modeled concentrations peak within a day and decay at the same rates as the data. Fit errors are most pronounced at the peaks of TNF-*α*, IL1, IL6, and IL10, and the tails of TNF-*α*, IFN-*γ*, IL1, and IL6. The concentration of IL12 is an order of magnitude smaller than that of the other cytokines.

**Figure 2:**
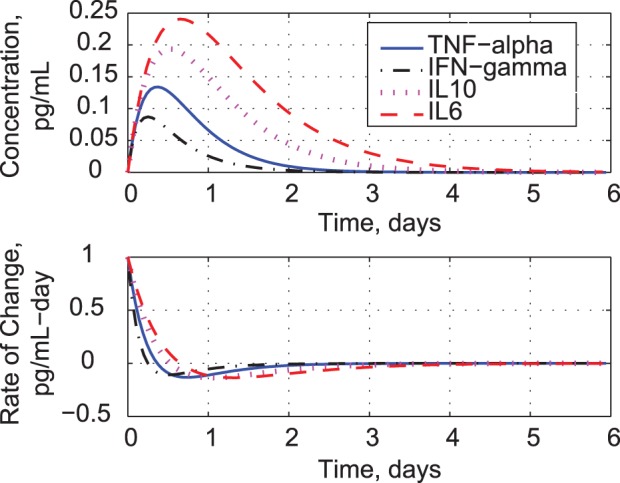
Response to unit initial rates of change for TNF-*α*, IFN-*γ*, IL10, and IL6.

**Figure 3:**
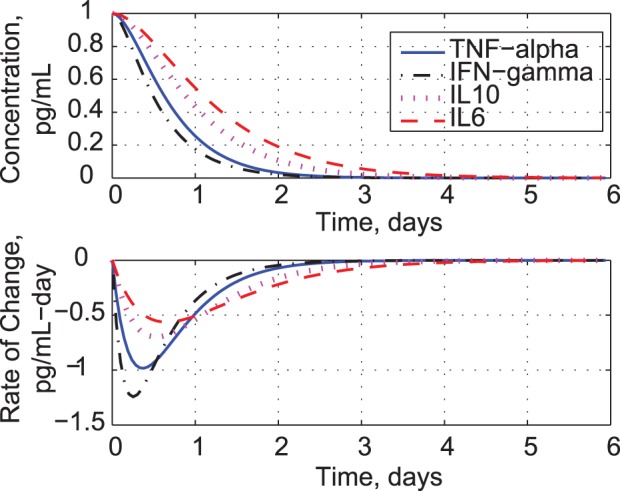
Response to unit initial concentrations for TNF-*α*, IFN-*γ*, IL10, and IL6.

The models allow us to predict responses to unit initial-conditions in concentration and rate of change. Because the models are linear, the response shapes are independent of initial-condition magnitude. Unit drug infusion is modeled as an impulsive force, producing initial rates of change in the cytokines (Fig. 2). Given equal starting points, IFN-*γ* ‘s rate is the quickest to rise and decay, and it achieves the lowest concentration peak. IL6 is the slowest to respond, but it reaches the highest peak. The IL6 concentration lingers for about four days, while the IFN-*γ* response is essentially over in two days. The decay in cytokine production rates correlates with the decrease in measured T-cell and monocyte populations [11]. Predicted responses to incremental changes in cytokine concentration are shown in Fig. 3. The relative speeds and magnitudes of each response are unchanged; however, the presence of two exponential modes is less obvious. The negative rates of change peak during the first day, while the concentrations decay smoothly to zero.

### An Integrated Model of Cytokine Response

#### Uncoupled Stability Matrix

The individual cytokine models provide the starting point for creating a single coupled model of response. The broad description of system dynamics (eq. 3b and 9) is unchanged, but the dimensions of the state vector, stability matrix, and state transition matrix are increased. Each cytokine is represented in the state vector by its concentration and rate of change:
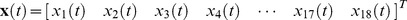
(12)


The odd components of **x**(*t_k_*) are concentrations, the even components of **x**(*t_k_*) are their rates of change, and dim(**x**) = (18×1). With no cytokine coupling, **A** is block-diagonal; its 18 coefficients are the same as those identified for the individual models.
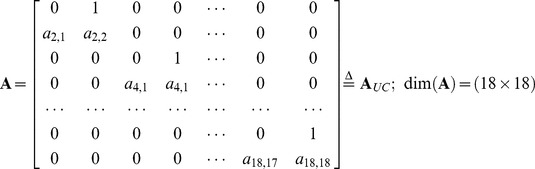
(13)


The corresponding state transition matrix, 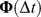, is,
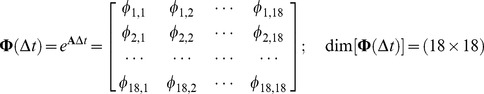
(14)where **A** *_UC_* denotes the uncoupled stability matrix and Δ*t* = 6 hr. The state transition matrix (eq. 14) propagates the state vector for evaluating the error cost, *J*, beginning with zero concentrations and the rates of change presented in Table 1:
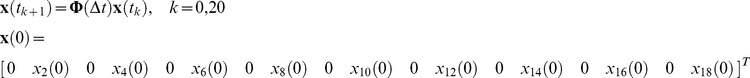
(15)


The error cost, *J*, is defined as,
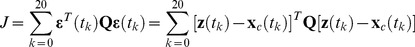
(16)where **z**(*t_k_*) is the (9×1) vector of clinical measurements at *t_k_*, and **x** *_c_*(*t_k_*) is the (9×1) vector of cytokine concentrations predicted by the model [i.e., the even components of **x**(*t_k_*)]. **ε**(*t_k_*) is the (9×1) difference between them at 21 time points. The (9×9) diagonal matrix, **Q**, normalizes the cytokine residuals to give them equivalent weight in the cost function:
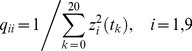
(17)


The approach is confirmed by applying the search algorithm to identify the uncoupled eighteenth-order model, **A** *_UC_*. The 27-parameter minimization reproduces the 18 eigenvalues and 9 initial rates of change of Table 1 to at least 3 significant digits.

### Coupled Stability Matrix

With cytokine coupling, each of the 72 off-diagonal (2×2) blocks of **A** contains a concentration sensitivity coefficient in its lower-left element:
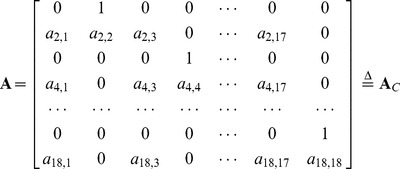
(18)


Fixing the initial rates of change at previous values (Table 1), the cost function is minimized with respect to 90 parameters: the 18 (*λ*_1_, *λ*_2_) parameters of the diagonal blocks and the 72 coupling parameters. The Nelder-Mead algorithm is robust, and it is guaranteed to find a local minimum of the cost function [26]. A positive-definite quadratic cost function with a LTI dynamic constraint theoretically possesses just one (global) minimum, but local minima may arise from numerical imprecision, e.g., finite word length, rounding, or truncation of computations. These local minima may be far removed from the global minimum of the unconstrained cost function (eq. 16). To assure that the minimization defines a coupled stability matrix, **A** *_C_*, in the neighborhood of **A** *_UC_*, we assign a small penalty to the sum of the squares of the off-diagonal parameters. This procedure is called *regularization*, and it is widely used in statistics and machine learning. Denoting the coupling parameters by the (72×1) vector, **p** *_C_*, a soft constraint weighted by *r_C_* is added to the error cost:
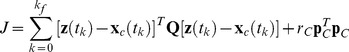
(19)


A large value of *r_C_* forces **p** *_C_* to zero, producing the same parameter vector as the uncoupled minimization. As the penalty weight is reduced, significant coupling parameters are revealed. When the penalty is several orders of magnitude smaller than the error cost specified by eq. 16, the **p** *_C_* estimate stabilizes and becomes insensitive to the value of *r_C_*.

The 90-parameter minimization reduces the error cost by 20%; however, the trace of the estimated **A** *_C_* is about 3% higher than the trace of **A** *_UC_*. (The trace of **A** equals the negative sum of its eigenvalues, and it is called the “total damping” of the system.) The coupling parameters should redistribute the eigenvalues but not change the total damping of the system model. Therefore, we penalize the square of the difference between the traces of **A** *_UC_* and **A** *_C_* in the augmented error cost function,
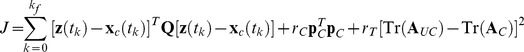
(20)where *r_T_* is a small coefficient. The trace penalty is several orders of magnitude smaller than the fit-error cost at the minimum. With its use, the difference in the traces is reduced to less than 0.1% and the fit-error cost is lowered by an additional 1%.

The odd columns and even rows of **A** *_C_* portray the sensitivities of cytokine response to cytokine concentration; they are presented as the (9×9) *concentration coefficient matrix*, **C** *_C_* (Table 2). The diagonal elements of the reduced matrix are negative, indicating stable self-regulation of each cytokine. The sense and magnitude of coupling effect that one cytokine has on another is given by each off-diagonal element. The even, diagonal terms of **A** *_C_* are negative, [−5.2, −8.6, −4.4, −8.0, −3.3, −8.1, −8.0, −5.5, −8.8], providing damping in the coupled system.

**Table 2:** Concentration Coefficients of the Fully Coupled Cytokine Model, C*_C_*.

	TNF	IFN	IL10	IL8	IL6	IL4	IL2	IL1	IL12
**TNF′′**	**−6.413**	0.345	**−0.383**	−0.186	**−0.632**	**−0.680**	−0.206	0.672	**−0.818**
**IFN′′**	−0.554	**−18.641**	0.078	**1.576**	**1.542**	0.128	0.184	**0.696**	**−0.903**
**IL10′′**	−0.487	**0.846**	**−3.320**	0.145	**−0.727**	−0.111	−0.030	−0.017	0.617
**IL8′′**	**0.992**	−0.207	**1.566**	**−13.571**	0.058	**−0.823**	−0.316	0.046	**−3.356**
**IL6′′**	0.412	**−1.688**	−0.303	0.042	**−2.784**	**0.640**	**0.769**	**0.955**	0.065
**IL4′′**	**−1.129**	**−1.072**	−0.278	0.271	0.101	**−16.305**	**0.776**	**0.778**	−0.237
**IL2′′**	−0.503	−0.775	**0.422**	**0.506**	−0.242	−0.022	**−15.226**	−0.181	−0.957
**IL1′′**	0.053	−0.090	−0.376	**0.891**	−0.575	0.227	0.289	**−7.571**	0.604
**IL12′′**	**−0.877**	−0.075	0.275	−0.228	0.320	0.343	**1.554**	−0.271	**−19.448**

Positive off-diagonal elements represent inductive acceleration of one cytokine by another; negative coefficients represent inhibitive acceleration. Input cytokines are listed in the first row. (.)” represents *d* ^2^(.)*/dt* ^2^ in the first column of the table.

**Figure 4:**
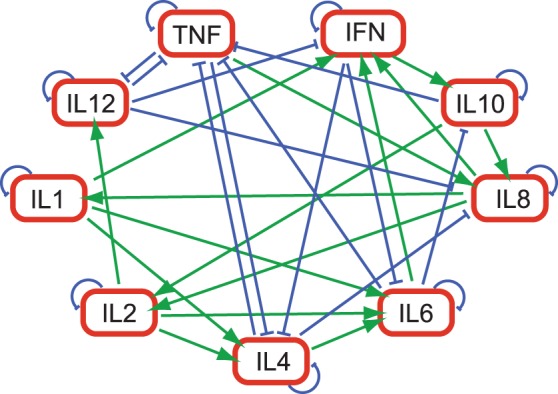
Most significant inductive and inhibitive accelerations in the cytokine coupling matrix. Arrowhead denotes induction; “T” represents inhibition.

Inductive and inhibitive effects are most readily visualized in Fig. 4, which illustrates the three highest-magnitude coupling paths for each cytokine, as well as the self-inhibition of each cytokine. There are three two-way paths in the figure. TNF-*α* mutually inhibits both IL4 and IL12, while IL6 enhances and is inhibited by IFN-*γ*. TNF-*α*, IFN-*γ*, and IL4 are involved in six strong coupling pathways, and IL6 and IL8 participate in seven. The three largest effects of IL1, IL2, and IL8 are inductive; the three largest effects of IL12 are inhibitory.

### Time Histories and Response Motifs

**Figure 5:**
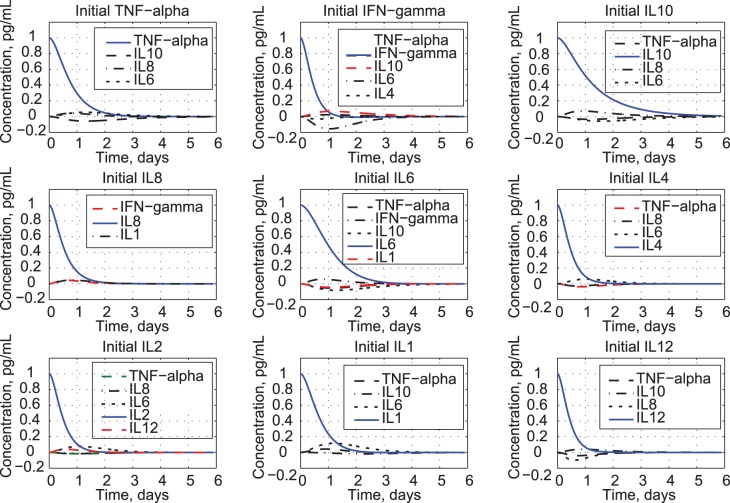
Unit initial-concentration response for nine cytokines based on the coupled.

Coupled responses to unit initial conditions are shown in Fig. 5. As each cytokine concentration decays, it excites responses in the remaining cytokines, whose initial values are taken to be zero. For clarity, only the most significant couplings are shown; nevertheless, the simulation includes all cytokines and their coupling effects. It is apparent that the coupling is small but significant. The pro-inflammatory cytokines TNF-*α*, and IL8, and IL1 induce other pro-inflammatory cytokines while down-regulating anti-inflammatory ones. Conversely, IL10 and IL4 perturbations inhibit TNF-*α*. The stimulated cytokines exhibit different time scales of response, with some peaking before others (e.g., responses to IL6, IL10, and IL12).

**Figure 6:**
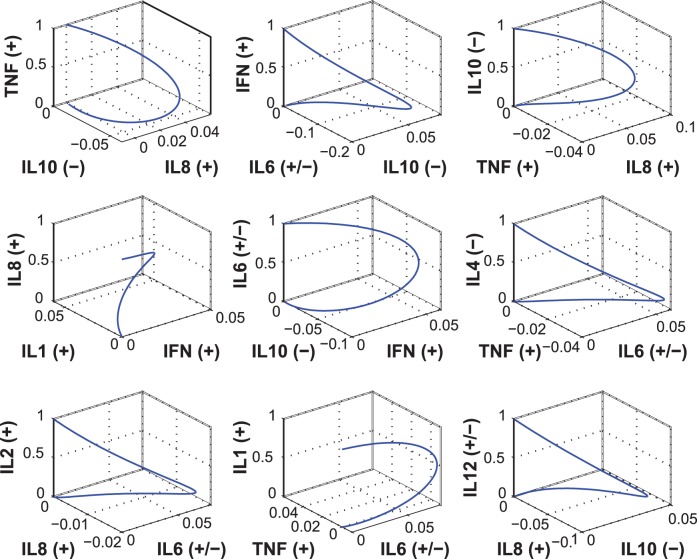
Motifs of response to unit initial cytokine concentrations.

The interplay among cytokines is demonstrated in Fig. 6, where the initialized cytokine is plotted on the vertical (*z*) axis, and the two most highly perturbed cytokines are plotted on the horizontal (*x* and *y*) axes. Thus the initial condition for each plot is (0, 0, 1), and, after five days, the state approaches (0, 0, 0). The symbols (+), (–), or (+/−) represent accepted pro-, anti-, or mixed-inflammatory classifications of each cytokine. These state-space plots portray motifs of the most significant cytokine responses. When the three cytokines respond on similar time scales, the curves are rounded; when the time scales are significantly different, the curves contain “hairpin” kinks. For example, an initial perturbation in IFN-*γ* stimulates a fast, small IL10 response with a slower and larger IL6 response, as verified by Fig. 5.

### Eigenvalues and Most Significant Eigenvector Components

The estimated stability matrix, **A** *_C_*, possesses four real and seven complex modes. Table 3 lists the modes in order of increasing eigenvalue magnitude. It presents the three most significant eigenvector component magnitudes for each mode, as well as the periods and damping ratios of the oscillatory modes. All cytokines are present in all eigenvectors; however, the third largest eigenvector components are typically much smaller than the first two. All of the oscillatory modes are heavily damped. Two pairs of modes, (5 and 6) and (8 and 9), have nearly identical periods, differing only in the smaller eigenvector components. Neither TNF-*α* nor IFN-*γ* is the largest component of any mode, although each is ranked second or third in several cases. IL2 appears only as the third component of Mode 8. IFN-*γ* and IL4 are equally represented in Mode 5, while IFN-*γ* and IL8 have equal representation in Mode 9. This suggests that these cytokines are most involved in inter-cytokine coupling. Modes 1 and 7 derive principally from IL10, while Modes 3 and 11 are largely due to IL8. Comparing the results to Table 1, IL4 and IL6 are seen to be the least impacted by inter-cytokine coupling.

**Table 3:** Eigenvalues, Periods, Damping Ratios, and Three Highest Eigenvector Magnitudes of A*_C_.*

Mode	*λ*, d^−1^	*P*, d	*ζ*, -	EV #1	EV #2	EV #3
**1**	–0.84	–	–	IL10	IL6	IL8
**2**	–1.4± j0.75	3.93	0.89	IL6	TNF	IL10
**3**	–1.88	–	–	IL8	TNF	IL1
**4**	–2.27± j0.61	2.66	0.97	IL1	IL8	IFN
**5**	–3.28± j0.60	1.89	0.98	IL1	IL10	IFN/IL4
**6**	–3.22± j0.98	1.86	0.96	IL1	IL4	TNF
**7**	–3.75	–	–	IL10	IL12	TNF
**8**	–4.02± j0.20	1.56	0.99	IL4	IL12	IL2
**9**	–4.41± j0.71	1.40	0.99	IL4	IL12	IFN/IL8
**10**	–5.29± j0.82	1.17	0.99	IL8	IFN	IL12
**11**	–5.82	–	–	IL8	IFN	IL12

### Principal Components of the Cytokine Response

**Figure 7:**
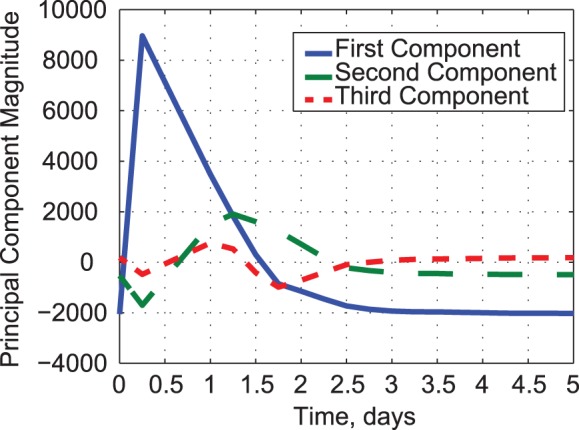
Shapes of the first three cytokine principal components, *y*_1_(*t_k_*), *y*_2_(*t_k_*), and *y*_3_(*t_k_*).

Principal components assess the similarity of wave shapes in each of the nine cytokine histories. They show orthogonal projections of the original data that are based on singular-value decomposition of the covariance matrix,/in (0, *t_f_*) [27], [28]. Unlike the modal analysis presented above, principal components do not address causality or coupling between dynamic variables; they are drawn together by the common stimulus, TGN1412. The (9 x 1) principal component vector, **y**(*t_k_*), can be expressed as.
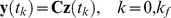
(21)where **C** is a square matrix derived from the singular values of **Z** that transforms the data, **z**(*t_k_*), into orthogonal components. *y*_1_(*t_k_*) is associated with the largest singular value of **Z**; hence, it describes the largest variability in the data set. *y*_2_(*t_k_*) describes the next highest, and so on. In the present case, the first principal component is correlated with 92% of the cytokine responses, while the next two components account for 7% of the correlation (Fig. 7). The remaining six principal components describe just 1% of the response shape similarities. All of the cytokines exhibit a single peak in growth and decay, as in *y*_1_(*t_k_*). Variations across the sample produce the two- and three-peak waves found in *y*_2_(*t_k_*) and *y*_3_(*t_k_*).

**Figure 8:**
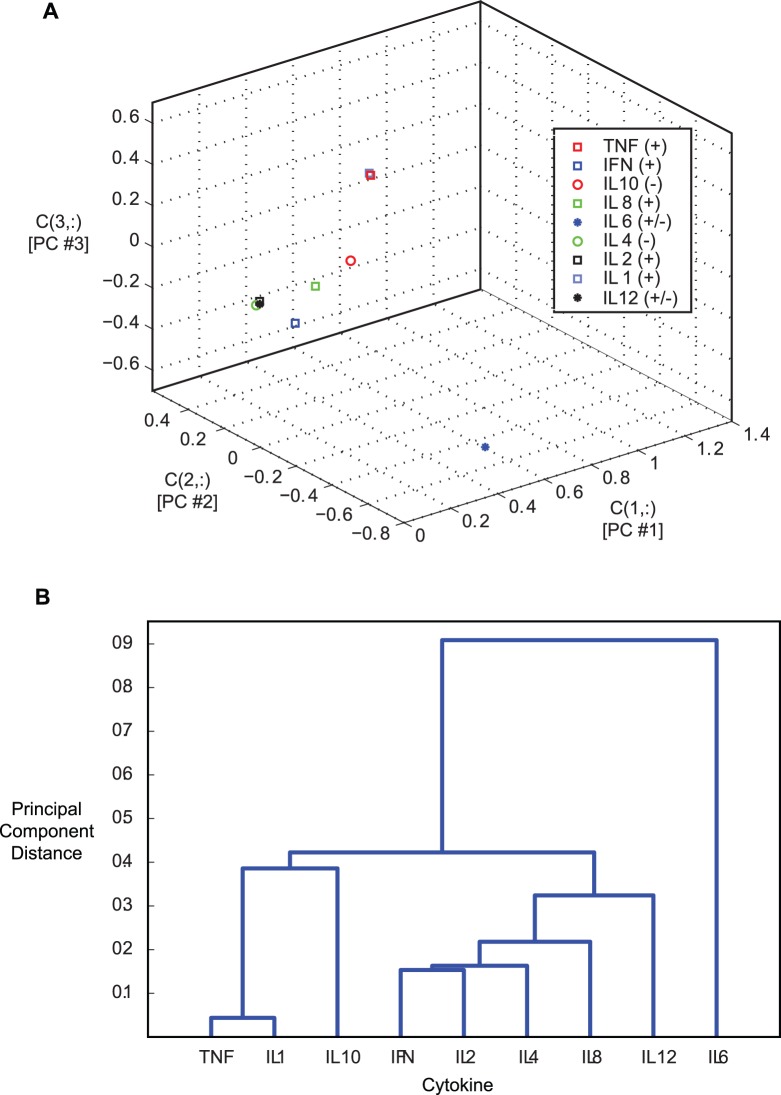
Similarity of cytokine response shapes as described by the first three principal component coefficients. A) Coefficients of the first three principal components. B) Dendrogram relating closeness of cytokine covariances.

The first three rows of **C** contain the coefficients for the largest principal components (Fig. 8A). The figure shows that the IL6 coefficients are far removed from those of the other eight cytokines. Two coefficient sets (TNF-*α* and IL1) overlie each other in the figure. Closeness connotes similar wave shape, but this three-dimensional depiction is ambiguous. The ambiguity is eliminated by computing the distances between coefficient triplets and linking their closeness in a tree, or *dendrogram* (Fig. 8B). The cytokines are grouped according to their distance from each other, with the height of the links indicating that distance. The dendrogram reveals that TNF-*α* and IL1 responses are indeed close and that IL10 response is similar to them. IFN-*γ*, IL2, and IL4 form a second cluster that also includes IL8 and IL12, while IL6 is separate from the rest.

**Figure 9:**
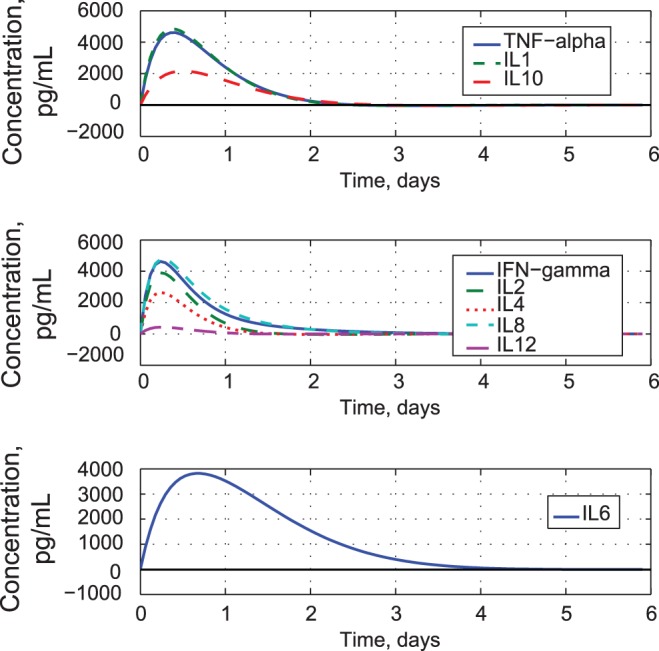
Modeled time histories of the three cytokine response groups with experimentally derived initial rates of change.

These groupings become apparent in the modeled cytokine response of Fig. 9. We see that TNF-*α* and IL1 concentrations are quite close; IL10 has similar shape but lower amplitude (Group A). IFN-*γ*, IL2, IL4, IL8, and IL12 are similar to each other (Group B) and have faster response than the first group. IL6 is in a class by itself, with slow response (Group C). Pro-inflammatory cytokines appear in Groups A and B, with IFN-*γ*, IL2, and IL8 in the faster group. IL4 is faster than the other anti-inflammatory cytokine (IL10), while mixed-category cytokines (IL6 and IL12) appear in the fastest and slowest groups.

### Applications of the Dynamic Cytokine Model

The mathematical model of cytokine-storm response allows us to investigate several additional topics that take advantage of the clinical trial data. The model can be used to study the effects of inhibiting individual cytokines, to predict the effects of different TGN1412 infusion rates, and to reveal effects of empirical uncertainty. These analyses are based on a linear model of a specific event, in which median cytokine concentrations are driven not only by TGN1412 and immune response but also by beneficial treatment in intensive care. There was considerable variation in the responses of the trial’s six subjects.

### Inhibiting Selected Cytokines

**Figure 10:**
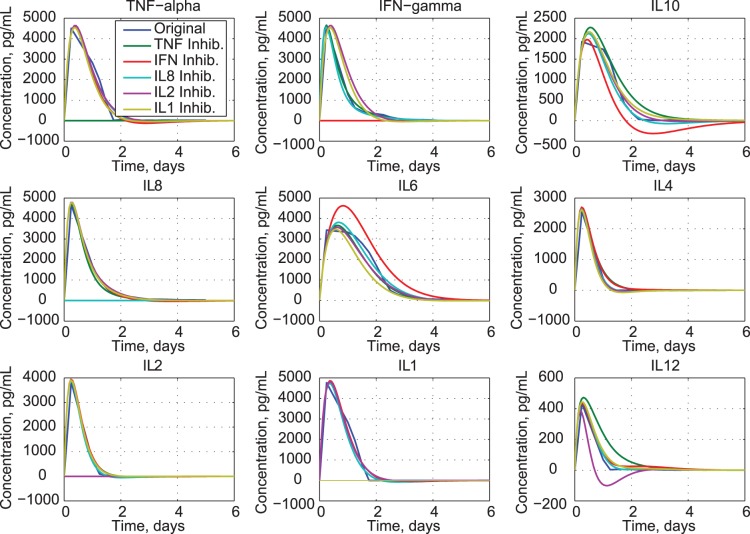
Effects of inhibiting pro-inflammatory cytokines.

The effects of inhibiting individual cytokines are revealed by solving the dynamic equation (eq. 3b) with requisite rows of **A** *_C_* (eq. 18) set to zero. For example, zeroing the first two rows eliminates all effects of TNF-*α*. Canceling individual effects provides an assessment of the significance of the missing variable’s dynamic coupling on the remaining eight cytokines. We examine missing cytokines in two sets: pro-inflammatory and anti−/mixed-inflammatory cytokines. Figure 10 shows that eliminating TNF-*α* has a weak effect on IL12, but little effect on other cytokines. The result suggests that TNF-*α* concentration is not a driving factor on the dynamics of the cytokine network. This is not surprising, given the variable role of TNF-*α* in immune cell biology [29]. Lack of IFN-*γ* induces more significant changes in IL10 and IL6. Inhibiting IL8 causes IL10 and IL12 to reach higher peaks and to decay more slowly. Losing IL2 produces an oscillatory response in IL12.

**Figure 11:**
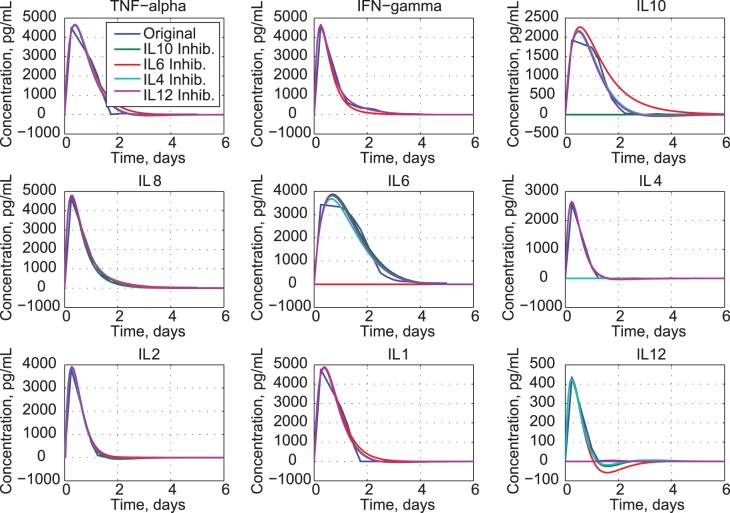
Effects of inhibiting anti- and mixed-inflammatory cytokines.

Inhibiting anti-inflammatory cytokines (IL10 and IL4) produces small effects on IL6 and IL12, with negligible effects on pro-inflammatory cytokines (Fig. 11). Loss of IL6 affects several cytokines, slowing the decay of TNF-*α*, IL10, and IL1, increasing the decay of IFN-*γ*, and producing oscillatory response in IL12. This result suggests that sustained presence of IL6 during the clinical trial has strong effect on the other cytokines. Eliminating IL12 does not significantly impact the other cytokines, in part because its response is an order of magnitude smaller than the others’ response.

### Effects of the Duration of TGN1412 Infusion

**Figure 12:**
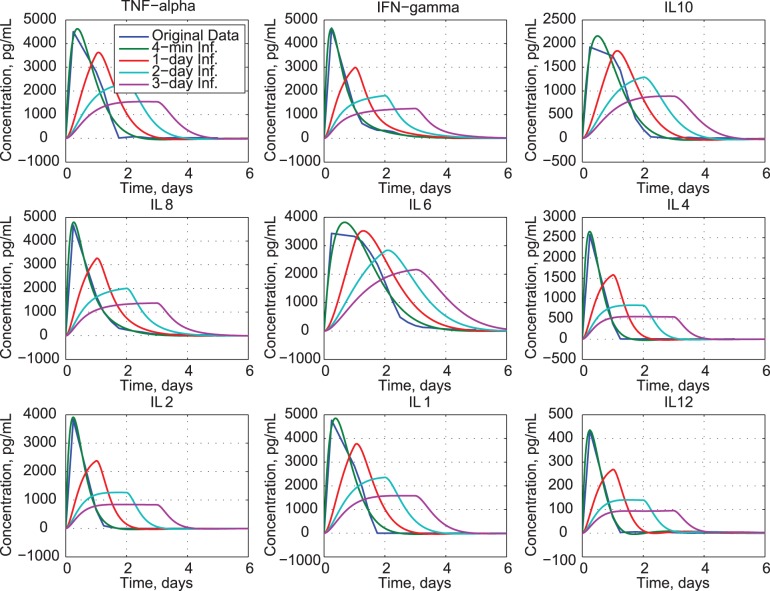
Effects of TGN1412 infusion duration of four minutes to three days on cytokine response. Dosage  = 8 mg.

The TGN1412 stimulus can be modeled explicitly by adding a forcing term to the dynamic equation,
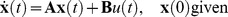
(22)and subsuming the previous initial rate of change in that term. The TGN1412 infusion rate is represented by *u*(*t*), and it affects cytokine propagation through the (18×1) matrix, **B**. The odd elements of **B** are zero, and the even elements are derived from the estimated initial rates (Table 1). Assuming that the clinical infusion lasts for 4 min and occurs at 2 mg/min or 2880 mg/day, B = (2880/8)x(0) = 360x(0). Maintaining a fixed dose of 8 mg, the TGN1412 infusion rate is inversely proportional to the infusion duration, *t_infusion_*. We see the results of increasing the infusion duration from 4 minutes to 3 days in Fig. 12, with pharmacokinetic effects neglected. The 4-min–infusion simulation is virtually identical to the results for instantaneous infusion. There is little change in profile for durations lasting several hours (not shown). For a one-day infusion duration at a rate of 0.0056 mg/min, several cytokine peaks remain close to their recorded values. Significant reduction in peak values requires infusion periods of 2–3 days, for which infusion rates are 1/720-1/960 of the trial values. However, stretching the infusion period prolongs the period when cytokine concentrations remain at unacceptably high levels. This model suggests that reducing infusion rate and increasing infusion period would have attenuated but not prevented the TGN1412 cytokine storms.

### Stochastic Effects on Cytokine Concentration

Observed cytokine concentrations are structured-but-random variables that vary from one patient to the next. Within each patient, cytokine levels are influenced by many signals other than TGN1412. Serum concentrations do not expose localized distributions within the body. Errors occur in sampling and analysis, and measurements are “snapshots” rather than continuous observations. Consequently, we can never know cytokine concentrations with deterministic accuracy. At best, we can estimate *probability distributions* of the concentrations and their rates. Probability distributions, whether Gaussian or not, are characterized to second order by their means and covariances [30]. We examine the propagation of these variables through the system equations derived in Sec. 3 and 4.

The mean vector,/, and covariance matrix, **P**(*t*), for an ensemble of multi-dimensional random variables, **x**(*t*), are.
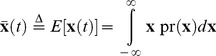
(23)

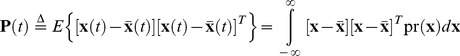
(24)pr(**x**) is the probability density function of **x** and *E*[.] denotes the expected value of the argument. Expected values can be propagated through time by dynamic models, given initial conditions and known inputs. Equation 22 is a deterministic equation when **A**, **x**(0), **B**, and *u*(*t*) are known exactly. It generates the expected (mean) value,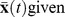, when the actual initial condition is unknown but has the mean value,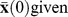, and *u*(*t*) is random. If *u*(*t*)  = 0 and **A** is known, the stochastic differential equation for the time-varying mean is
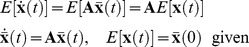
(25)


The (18×1) cytokine mean vector is propagated over discrete intervals of time, Δ*t*, by.
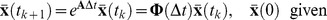
(26)


Cytokine covariance is propagated as the expectation of the outer product of eq. 26. With no uncertain forcing, the (18×18) covariance matrix estimate, **P**(*t_k_*), depends only on its initial condition,
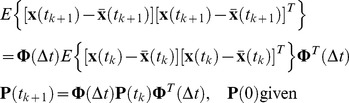
(27)


The diagonal elements of **P**(*t_k_*) are the estimated variances of **x**(*t_k_*), and their square roots are the corresponding standard deviations, 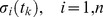. *x_i_*(*t_k_*) and *σ_i_*(*t_k_*) have the same units; plotting [*x_i_*(*t_k_*) ± *σ_i_*(*t_k_*)] vs. *t_k_* would produce an envelope containing ∼68% of probable responses if the random variables were Gaussian.

**Figure 13:**
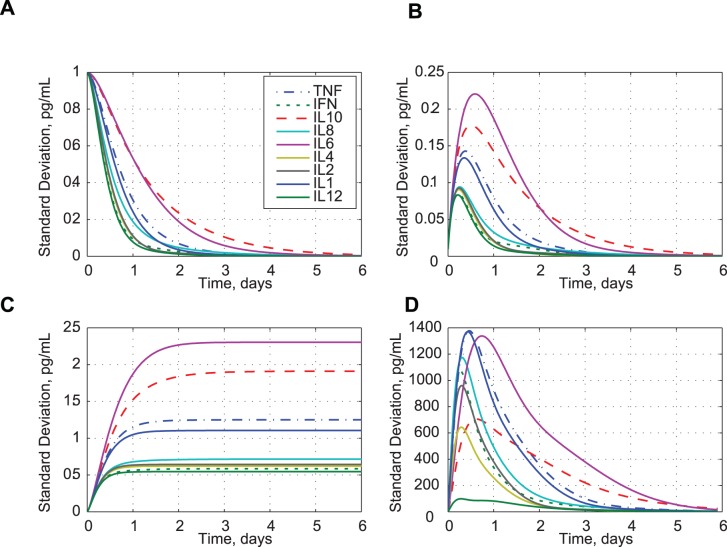
Effects of uncertainty in initial conditions, random disturbances, and system parameter variations on cytokine standard deviations. A) Standard deviation with initial concentration uncertainty. B) Standard deviation with initial rate uncertainty. C) Standard deviation with process noise. D) Standard deviation with parameter variations.

Choosing **P**(0) to be a diagonal matrix allows us to estimate how initial uncertainties in the cytokines and their rates would propagate during the cytokine storm (Fig. 13). For these examples, we assume that the computation interval, Δ*t*, is 0.01 days. With unit initial variances in all concentrations (zero even elements and ones in the odd elements of **P**(0)), the standard deviations decay as seen in Fig. 13A. Unit initial rate variances (zero odd elements and ones in the even elements of **P**(0)) produce the concentration standard deviations shown in Fig. 13B. Equations 25 and 27 are linear in **x**(*t_k_*) and **P**(*t_k_*); the standard deviation responses are similar but not identical to the cytokine mean value responses (Fig. 1–4). Initial uncertainties in IL 6 and IL10 are the slowest to decay, while those in IL12 and IFN-*γ* are the fastest.

To assess the effects of continued random forcing of the cytokines by elements other than TGN1412 (e.g., unspecified immunological response), we add an (18×18) disturbance covariance, **W**, to eq. 27 and set the initial error covariance matrix to zero:
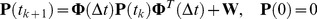
(28)


For illustration, **W** is taken to be a diagonal forcing matrix with zero odd elements and ones in the even elements. The cytokine standard deviations reach steady values after several days (Fig. 13C), modeling quasi-homeostatic differences in cytokine levels. The ratios of these levels are of interest because they are only loosely related to TGN1412 stimulation. IL6 and IL10 concentrations show the largest continuing uncertainty, while IL12 and IFN-*γ* are the lowest.

Small random uncertainties in stability matrix coefficients can be assessed within the same format. The effects of unit variances in the damping elements of **A** *_C_*, *a*_2*i*,2*i*_, *i* = 1,9, are shown in Fig. 13D. Following [30], the disturbance covariance, **W**(*t_k_*), takes the form,
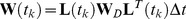
(29)where **L**(*t_k_*) contains the elements of **x**(*t_k_*) in (0, *t_f_*) on its main diagonal and **W** *_D_* is a diagonal matrix with ones in the even terms and zeros in the odd terms. The assumed parameter uncertainties are typically 10–25% of the nominal parameter values; hence, the response effects are more pronounced than those of the other three uncertainty examples. Figure 13 shows that IL1, IL6, and TNF-*α* have higher response to these parameter uncertainties, while IL4, IL10, and IL12 have less.

## Discussion

The TGN1412 clinical trial provides a narrow window through which to assess causal and coordinated associations of cytokines during a drug-induced “storm.” It allows us to assess the relative responses of nine important signaling molecules and to infer their inter-regulatory effects. Cytokine concentrations are expected to respond (via cellular intermediaries) to the TGN1412 stimulus, to the aggressive immunosuppressive treatment that the patients received, and to coupling effects among cytokines. The cytokine response induced by TGN1412 and by treatment cannot be separated in the present data. However, coupling among cytokines and sensitivity of the dynamics to removal of individual cytokines can nonetheless be explored.

While cytokines are associated principally with the immune system, they also are linked to epithelial and endothelial cells, smooth muscle, and adipose tissue. Thus, it is not surprising that clinical signs included headaches, muscle pain, nausea, diarrhea, vasodilation, and hypotension. When recruited, immune cells secrete cytokines, and cytokines regulate cellular activity; thus, it is of interest to examine possible underlying cellular relationships from the cytokine profiles. Additional laboratory results shown in [11] can be compared to the principal components of cytokine response. Cytokine Group B [IFN-*γ*, IL2, IL4, IL8, and IL12] had the fastest response to TGN1412 (Fig. 9), peaking about six hours after TGN infusion. It was during this time that T cell, monocyte, and platelet concentrations crashed. While cytokine production depends on the concentration of cells that secrete them as well as per-cell secretion rates, a sudden burst of cytokine production by activated cells could lead to their apoptotic death and to reduced cell populations. Group B cytokines returned to near basal levels after two days, at which point these cells were recovering to normal values.

The neutrophil profile is reminiscent of the slower IL6 (Group C) response, peaking 1–2 days after infusion; modeled neutrophil concentration remains above its upper reference range for more than 10 days in response to the systemic damage caused by the event. The growth and decay of serum creatinine, which is elevated during renal dysfunction, occurred on a time scale that parallels Group A [TNF-*α*, IL1, and IL10] response. C-reactive protein concentration, a strong indicator of inflammation, peaks 2–3 days after infusion, on a slower time scale than any of the cytokine groups. It returns to normal levels about 10 days after the event.

Cells that secrete the subject cytokines and that are regulated by them are listed in Table 4, with S denoting “secreted by” and R indicating “regulated by”. This table is based on a literature search [3], [4], [5], [6], [21], [29], [31], [32], [33], [34], [35], [36], [37], [38], [39], [40], [41], [42], [43], [44]. Cells of the innate and adaptive immune systems are involved in all phases of the cytokine storm that was induced by TGN1412. IL8, which is a pro-inflammatory member of the fast Group B, is not identified as interacting with adaptive cells. It is associated with the innate immune system (monocyte, macrophage, neutrophil, dendritic and mast cells), as well as several tissue groups, and [44] indicates that it is the only cytokine secreted by neutrophils. Excepting their association with IL4, IL8, and IL12, innate immune cells and “other” tissue types are underrepresented in Group B, appearing primarily in the slower Groups A and C.

**Table 4:** Cells That Secrete and are Regulated by the Measured Cytokines.

	Group A			Group B					Group C
	TNF-α	IL1	IL10	IFN-γ	IL2	IL4	IL8	IL12	IL6
Innate System									
Monocyte	S	S	S	R	R		R		S, R
Macrophage	S, R	S, R	S, R	R		R	S, R	S	S
Dendritic Cell	S, R	S	S, R	S, R	S	R	S	S	
Mast Cell	S	S, R	S	S	S	S	S, R		S
Neutrophil	S, R	S, R	R			S	S, R	S	S, R
Eosinophil	S	S, R	S	S		S, R		S	S
Basophil	S	S, R		R		S			S
NK	S, R	S, R	S, R	S	S, R	S, R		R	
Adaptive System									
B	R	S, R	S, R	S, R	R	S, R		S, R	S, R
Th1	S, R	S, R	S, R	S, R	S, R	S, R		S, R	S, R
Th2	S, R	S, R	S, R	S, R	S, R	S, R		S, R	S, R
CTL	S, R	S, R	S, R	S, R	S, R	S		S, R	S, R
									
Other									
Fibroblast	S, R	S, R		R			S		S
Epithelial Cell	S	S	S	R			S, R		S, R
Endothelial Cell	S, R	S, R		R			S, R		S, R
Smooth Muscle	S, R	S, R		R		S	S		S, R
Adipose Tissue	S, R	S							S, R

Cell types that secrete the cytokine are denoted by S; those that are regulated by the cytokine are indicated by R.

Further insights about cytokine dynamics could be gained by additional empirical trials involving the drugs that were used to treat the TGN1412 cytokine storm. These drugs – corticosteroids, chlorpheniramine, acetaminophen, ondansetron, metaraminol, methylprednisolene, and the anti-IL2 receptor antagonist antibody – could be administered safely to healthy subjects at low dosages for short periods of time. Cytokine concentrations would be measured at regular intervals, and the analysis presented in this paper could be applied to the data. Ancillary data, including clinical metrics, could be collected in the process. Unlike pharmacologic clinical trials focused on the safety or efficacy of new drugs, the empirical trials would be directed at gaining new knowledge about fundamental biology. Cross comparisons would allow the cytokine coupling effects to be distinguished from the direct effects of the drug stimuli, in a secure, well-controlled environment. This approach could lead the way toward developing a strong theoretical basis not only for understanding cytokine storms but for explaining many aspects of human physiology.

### Conclusion

The cytokine storm event produced by the 2006 Clinical Trial of TGN1412 can be simulated well by an eighteenth-order, linear, time-invariant dynamic system. Each cytokine’s response is assumed to be proportional to its current concentration and its current rate of change; thus, its response is represented by a second-order differential equation. Nine interacting cytokines are, therefore, represented by an eighteenth-order system. Coefficients of the model are found by minimizing an error cost function using the downhill-simplex method. The system model provides evidence for the regulation of cytokines by other cytokines, identifying inductive and inhibitive relationships among the nine cytokines as well as similarities in temporal histories.

The present analysis illustrates that reducing the dosage rate of TGN1412 while increasing the duration of infusion (i.e., for fixed total dosage), would have little effect on peak cytokine concentrations until the infusion duration exceeded one day. This effect would not be beneficial, as the period during which cytokines were at unacceptably high levels also would increase. This suggests that the drug, not the dose used during the trial, was the crux of the problem. Analysis of the data’s principal components reveals that cytokine response profiles fall into three groups: [TNF-*α*, IL1, and IL10], [IFN-*γ*, IL2, IL4, IL8, and IL12], and [IL6]. Association of these cytokines with cellular secretion and regulation suggest that the adaptive immune system had a dominant effect in the cytokine storm (perhaps unsurprising, given the source of the stimulus). As suggested by the literature review, the pro-inflammatory cytokine, IL8, was most likely produced by innate system cells and non-immune tissue.

This paper presents a sequence of general analytical procedures that are useful for interpreting temporal biomedical data for a wide variety of systems. Time constants, natural frequencies, damping ratios, and mode shapes of response modes are estimated, response to initial conditions other than those of the clinical trial are predicted, and stochastic effects are assessed. It is envisioned that extensions of this analysis to empirical biological trials using safe pharmaceutical agents to stimulate dynamic response could establish new paradigms in the mathematical theory of biology.
